# Highly Porous Titanium Cups Frequently Presenting with Radiolucent Lines in Cementless Primary Total Hip Arthroplasty: A Retrospective Cohort Study

**DOI:** 10.3390/jcm13113297

**Published:** 2024-06-03

**Authors:** Yoichi Ohta, Ryo Sugama, Yukihide Minoda, Shigekazu Mizokawa, Shinji Takahashi, Mitsuhiko Ikebuchi, Tamotsu Nakatsuchi, Hiroaki Nakamura

**Affiliations:** 1 Department of Orthopaedic Surgery, Osaka Metropolitan University Graduate School of Medicine, 1-4-3, Asahimachi, Abeno-ku, Osaka 545-8585, Japan; sugama@omu.ac.jp (R.S.); yminoda@omu.ac.jp (Y.M.); mizokawa-s@omu.ac.jp (S.M.); stakahashi@omu.ac.jp (S.T.); s21841j@omu.ac.jp (M.I.); hnakamura@omu.ac.jp (H.N.); 2 Tsuji-geka Rehabilitation Hospital, 3-24 Ikutamamaemachi, Tennnouji-ku, Osaka 543-0072, Japan; nakatsuchi@kankikai.com

**Keywords:** hip, surgery, primary total hip arthroplasty, highly porous titanium cup

## Abstract

**Background/Objectives:** A highly porous titanium cup with a three-dimensional metal interface was recently introduced to improve biological fixation and survival. However, radiography has revealed concerns regarding these cups, despite their excellent short- and mid-term clinical outcomes. This study compared the clinical and radiographic results of a highly porous titanium cup with those of a hydroxyapatite-coated porous titanium cup after primary total hip arthroplasty (THA). **Methods:** Fifty-one primary THAs were investigated. A highly porous titanium cup was used in 17 hips, and a hydroxyapatite-coated porous titanium cup was used in 34 hips. No significant differences in preoperative patient demographic characteristics were observed between the two groups. The 2-year postoperative clinical and radiographic results were compared. **Results:** Radiolucent lines were observed in 13 (76%) of 17 hips with highly porous titanium cups and in none (0%) of 34 hips with hydroxyapatite-coated porous titanium cups (*p* < 0.001). In the highly porous titanium cup group, radiolucent lines were observed in five hips (29%) in one zone, two hips (11%) in two zones, and six hips (35%) in three zones. No cup loosening was observed in either group. **Conclusions:** Radiolucent lines were significantly more frequent in highly porous titanium cups. This study suggests that, compared to the three-dimensional structure of porous titanium, the hydroxyapatite coating of porous titanium had a greater influence on bone ingrowth in the short term. The meaning of these findings in the long-term is unclear yet.

## 1. Introduction

Total hip arthroplasty (THA) is one of the most popular orthopedic surgeries, with high postoperative patient satisfaction. The number of THA surgeries is increasing yearly worldwide and is expected to increase further [1,2,3,4,5]. Cementless acetabular cup fixation is one of the most important factors affecting the long-term durability of primary THA. Porous tantalum metal acetabular components, which have been introduced to improve osseointegration and survivorship, can be used to obtain stable fixation with excellent long-term clinical results [6,7,8,9,10]. To improve biological fixation and survival, several companies have introduced and manufactured highly porous titanium cups with a three-dimensional metal interface. Several types of highly porous titanium cups have been clinically evaluated. Perticarini et al. reported the mid-term clinical performance of the trabecular titanium acetabular component, the DELTA-TT cup (Lima Corporate, Villanova di San Daniele del Friuli, Italy) [11]. Assaf et al. outlined mid-term clinical results for an R3 acetabular cup with Polarstem (Smith & Nephew, Memphis, TN, USA) [12]. Tamaki et al. described excellent clinical outcomes with a highly porous OsseoTi titanium cup (Zimmer Biomet, Warsaw, IN, USA) at ≥2 years postoperatively [13]. Kaneko et al. recorded excellent short- to mid-term clinical outcomes using the SQRUM TT cup (Kyocera Medical, Kyoto, Japan) [14]. Naziri et al. reported excellent short- and mid-term clinical outcomes with the Tritanium cup [15]. Carli et al. conducted short- to mid-term follow-up comparative studies of the Tritanium cup [16], and Yoshioka et al. presented the results of a short-term clinical and radiological comparative study of the Tritanium cup (Stryker Orthopaedics, Mahwah, NJ, USA) [17]. Despite good clinical results, there are concerns regarding the radiographic findings of radiolucent lines associated with some highly porous titanium cups [13,14,16,17].

Therefore, we hypothesized that there would be concerns regarding the postoperative radiological outcomes of highly porous titanium cups used in our institute. This study aimed to compare the clinical and radiographic outcomes of a highly porous titanium cup with those of a hydroxyapatite (HA)-coated porous titanium cup after primary THA.

## 2. Materials and Methods

### 2.1. Study Design

We conducted a retrospective cohort study of 51 primary THAs performed at our institution. The study included patients who underwent primary THA using a highly porous titanium cup (Tritanium hemispherical cup; Stryker Orthopaedics, Mahwah, NJ, USA) or HA-coated porous titanium cup (Trident HA hemispherical cup; Stryker Orthopaedics, Mahwah, NJ. USA). A highly porous titanium cup was used in 17 hips, and an HA-coated porous titanium cup was used in 34 hips. Patients who underwent revision THA or had a diagnosis of infection were excluded from the study. This study was approved by the Institutional Review Board of Osaka City University (approval number: 1280). As this was a retrospective study, the opt-out method was used instead of obtaining informed consent.

### 2.2. Surgical Procedure

The surgeries were performed by three senior joint surgeons who were experienced in the use of these titanium cups and were in the same surgical team. The cup choice was determined according to the surgeon’s preference. In the highly porous titanium cups, 14 hips were under-reamed by 1 mm, two hips were under-reamed by 2 mm, and one hip was reamed to the same size. In the HA-coated porous titanium cups, 30 hips were under-reamed by 1 mm, and four hips were under-reamed by 2 mm. In the highly porous titanium cup group, all hips used cluster hole cups. In contrast, 31 hips in the HA-coated porous titanium cup group used cluster hole cups and three hips used multihole cups. Screw fixation was performed to increase the initial stability, as desired by the surgeon. Screws were used in 4 of 17 highly porous titanium cups and in 8 of 34 HA-coated porous titanium cups. The number of screws used ranged from one to three at the surgeon’s discretion. Highly cross-linked polyethylene inserts (Trident X3: Stryker Orthopaedics, Mahwah, NJ, USA) were placed in all cups of both groups.

### 2.3. Clinical and Radiographic Evaluation

Demographic, clinical, and radiographic outcomes were reviewed from medical records and imaging reports. Preoperative clinical data included age, sex, height, weight, body mass index, lumbar bone mineral density (BMD), and diagnosis. The 2-year postoperative clinical and radiographic results of the highly porous and HA-coated porous titanium cups were compared. Functional evaluations were based on the modified Harris hip score (HHS) and were performed preoperatively and 2 years postoperatively. On postoperative anteroposterior radiography of the pelvis, we assessed the presence of radiolucent lines in the three radiographic zones as described by DeLee and Charnley [18], the fixation grade as per the modified DeLee and Charnley classification described by McPherson et al. [19], and evidence of aseptic loosening. The presence of a complete radiolucent line ≥ 2 mm around the acetabular cup in the three radiographic zones was considered a defining radiographic criterion for aseptic loosening. All radiographic measurements were performed by an experienced orthopedic surgeon who was not one of the operating surgeons.

### 2.4. Statistical Analysis

Qualitative variables were compared using the chi-square test or Fisher’s exact test. Continuous variables were analyzed using the *t*-test. Statistical significance was set at *p* < 0.05. All *p*-values were two-sided. Continuous variables were tested for normality using the Shapiro–Wilk test. All analyses were performed using SAS version 9.4 (SAS Institute Inc., Cary, NC, USA).

## 3. Results

Table 1 shows the preoperative demographic data and diagnoses. There were three men and 14 women in the highly porous titanium cup group, and 5 men and 29 women in the HA-coated porous titanium cup group. The mean participant ages were 63.8 ± 15.7 and 67.0 ± 14.9 years in the highly porous and HA-coated porous titanium cup groups, respectively. The mean heights were 153.1 ± 10.2 and 152.7 ± 8.3 cm, respectively; the mean weights were 56.2 ± 11.5 and 54.6 ± 9.7 kg, respectively; and the mean body mass indexes were 23.9 ± 3.8 and 23.5 ± 4.2 kg/m^2^, respectively. There were no significant between-group differences in the demographic parameters. The mean preoperative lumbar spine BMD was –1.6 ± 1.7 and –0.6 ± 1.9 in the highly porous and HA-coated porous titanium cup groups, respectively. There were no significant between-group differences in preoperative lumbar spine BMD (Table 1). Regarding the diagnosis, 10 patients had osteoarthritis, and seven had osteonecrosis of the femoral head in the highly porous titanium cup group. Twenty-eight patients had osteoarthritis, five had osteonecrosis of the femoral head, and one had an insufficiency fracture in the HA-coated porous titanium cup group.

The mean preoperative modified HHS was 44.5 ± 12.5 and 47.2 ± 10.3 in the highly porous and HA-coated porous titanium cup groups, respectively. There were no significant between-group differences in the preoperative modified HHS scores. The mean postoperative modified HHS at 2 years was 89.3 ± 7.4 and 92.3 ± 6.6, respectively. The modified HHS score improved significantly at 2 years in both groups (*p* < 0.01; Table 2).

In the immediate postoperative period, no radiolucent lines were observed around the cups in either the highly porous or HA-coated porous titanium cup groups. At the 2-year follow-up, radiographic analysis indicated that 13 highly porous titanium cups (76%) presented with radiolucent lines: five (29%) in one zone, two (11%) in two zones, and six (35%) in all three zones (Table 3). No radiolucent lines were observed in the HA-coated porous titanium cups (Table 3; *p* < 0.001). Screws were used in only four highly porous titanium cups; three of the four cups showed radiolucent lines. However, 10 of the 13 highly porous titanium cups without screws had radiolucent lines. No significant differences in the presence of radiolucent lines were found between the highly porous titanium cups with and without screws (*p* = 1.000). In accordance with the classification described by McPherson et al. [19], six hips (35%) had stable fibrous fixation, and 11 hips had bone ingrowth in the highly porous titanium cup group. All hips in the HA-coated porous titanium cup group exhibited bone ingrowth. No evidence of vertical or horizontal migration or of cup loosening was observed in either group in the immediate postoperative period or at 2 years postoperatively.

## 4. Discussion

Currently, various highly porous acetabular cups have been introduced and are commercially available. Macheras et al. reported 99% survival in a porous tantalum trabecular metal acetabular cup in 151 hips at the 8-year follow-up [6]. Naziri et al. reported the absence of progressive radiolucency or cup migration in 252 patients who received highly porous titanium cups using Tritanium cups at a mean follow-up of 36 months [15]. Perticarini et al. reported no radiolucent lines or periprosthetic osteolysis and a 99.3% survivorship for the trabecular titanium acetabular component using DELTA-TT cups in both primary and revision THA at the 5-year follow-up [11]. Assaf et al. reported excellent mid-term clinical results of the R3 acetabular cup with Polarstem, with a survivorship of 97.69% at 7 years. They noted no radiolucency in the R3 acetabular cup [12]. Kaneko et al. reported excellent short- to mid-term clinical outcomes using the SQRUM TT cup (Kyocera Medical, Japan). They also reported that radiolucent lines were seen in one DeLee and Charnley zone in 10 of 62 (16.1%) hips, in two zones in 10 of 62 (16.1%) hips, and in three zones in 1 of 62 (1.6%) hips in younger patients (age < 80 years). Radiolucent lines were observed in one zone in 9 of 42 (21.4%) hips, in two zones in 6 of 42 (14.3%) hips, and in three zones in 1 of 42 (2.4%) hips in older patients (age ≥ 80 years) [14]. Comparative studies on highly porous titanium cups have reported radiographic and clinical outcomes of primary THA. Tamaki et al. reported excellent clinical outcomes with a highly porous OsseoTi titanium cup (Zimmer Biomet, Warsaw, IN, USA) for at least 2 years postoperatively. However, the incidence of radiolucent lines was higher in the OsseoTi titanium cup than in the HA-coated titanium cup (Trident). The incidence of radiolucent lines in the OsseoTi titanium cup group was 22.8% at 24 months. Radiolucent lines were noted in one DeLee and Charnley zone in 24 of 101 (23.8%) hips and in two zones in 12 of 101 (11.9%) hips [13]. Naziri et al. reported excellent short- and mid-term clinical outcomes with the Tritanium cup [15]. Vutescu et al. conducted a comparative survival analysis of trabecular metal cups. They concluded that both the Tritanium and trabecular metal cups had excellent survival at the mid-term follow-up [20]. Palomäki et al. reported that the Tritanium cup had an increased risk of revision for any reason compared with conventional titanium alloy cups, using data from the Finnish Arthroplasty Register. Comparing the two groups with regard to revision for any reason, the 5-year Kaplan–Meier survivorship of the Tritanium cup group (94.7%) was significantly inferior to that of the conventional titanium alloy cup group (96.0%). In the Cox regression analysis, there was an increased risk of revision for any reason in the Tritanium cup group compared with the conventional titanium alloy cup group at >4 years (hazard ratio, 3.12) [21].

This study reports the short-term radiographic and clinical outcomes of highly porous titanium cups compared to those of HA-coated porous titanium cups. We found no evidence of cup aseptic loosening and obtained excellent clinical results in the highly porous titanium cup group, which is in agreement with the clinical outcomes reported by Naziri et al. [15]. However, radiographic examination revealed radiolucent lines around the cup in 76% of the patients in the highly porous titanium cup group. Circumferential radiolucent lines in all three zones were observed in 35% of the patients. Carli et al. reported that Tritanium primary cups after ≥2 years of follow-up revealed radiolucent lines in two or more DeLee and Charnley zones in 35 of 94 (37.2%) cups and in three zones in 18 of 94 (19.1%) cups [16]. Moreover, 24 of 70 (40.0%) Tritanium primary cups exhibited radiolucency in two or more zones, and 12 of 70 cups (17.1%) recorded radiolucency in three zones at ≥5 years of follow-up [16]. Yoshioka et al. reported that radiolucent lines were noted in 33 of 130 (25.3%) Tritanium cups in one DeLee and Charnley zone, in 29 of 130 (22.3%) cups in two zones, and in 17 of 130 (13.0%) cups in all three zones within an average follow-up period of 41.3 months [17]. Bingham et al. reported that 32 of 102 (31%) Tritanium cups had radiolucency in two or more DeLee and Charnley zones in ≥1 year of follow-up [22].

In contrast, radiographic analysis of HA-coated porous titanium cups (Trident) revealed no radiolucency around the cups. Wang et al. reported the clinical and radiological findings of 613 primary THAs implanted in HA-coated porous titanium cups within a minimum of 2 years of follow-up. They reported excellent clinical and radiographic results, no radiolucency, and no evidence of acetabular loosening [23]. The clinical outcomes of the HA-coated porous titanium cup in the current study were excellent and in agreement with those reported by Wang et al. [23]. Moilanen et al. reported the radiological outcomes of acetabular components coated with or without HA at the 2- to 3-year follow-up. They described fewer radiolucent lines on radiographs of cups coated with HA [24]. Tamaki et al. evaluated the radiological findings of 100 Trident cups at 2 years of follow-up. They reported that no radiolucent line was observed in Trident cups within the follow-up period [13]. Carli et al. reported that Trident peripheral self-locking (PSL) HA acetabular cups revealed no radiolucent lines in two or more DeLee and Charnley zones (0 of 98) after ≥2 years of follow-up [16]. Moreover, they reported that no radiolucent lines were observed in Trident PSL cups (0 of 19) after more than 5 years [16].

The most important finding of this comparative study was that the number of patients with radiolucent lines was significantly higher in the highly porous titanium cup group. According to the manufacturer’s information, to increase the initial stability, the coefficient of friction of the highly porous titanium cup (Tritanium) should be 1.01, which is higher than that of the HA-coated porous titanium cup (Trident) (0.81). Young’s modulus of the highly porous titanium cups is 113 GPa at the shell and 106 GPa at the porous surface. These data are equal to those of the HA-coated porous titanium cup, although the width of the shell varied between the highly porous titanium cups (4.8–5.8 mm) and the HA-coated porous titanium cup (3.3–4.3 mm). Therefore, the HA-coated porous titanium cup may be more flexible than the highly porous titanium cup. Another important difference between the two cups was the structure of the cup surface. The HA-coated porous titanium cup in this study had a porous structure created using the arc deposition method with an HA coating. The highly porous titanium cup has a three-dimensional highly porous structure created using the particle-sintered foam method without HA coating. From these perspectives, an HA-coated porous titanium structure may have a salutary influence on bone ongrowth or ingrowth of cementless acetabular cups compared with a highly porous titanium structure. Carli et al. and Long et al. speculated that the radiographic findings of the Tritanium cup were possibly caused by the manufacturing process of the Tritanium primary component surface, such as the pore structure and binding agent [16,25]. Yoshioka et al. reported differences in metal properties and elastic moduli between titanium and tantalum regarding the appearance of radiolucent lines [17]. Tamaki et al. mentioned the porous structure of the Tritanium cup, which is created by particle-sintered foam coating [13]. They noted the risk of sacrificial pore formers or proprietary binders remaining on the cup surface, which may have a detrimental effect on osseointegration [13].

Bingham et al. reported that only 8% of patients with adjuvant screw fixation in the Tritanium group demonstrated radiolucency compared to 45% of patients without adjuvant screw fixation [22]. They also stated that the Tritanium primary cup may be less suitable and more prone to fibrous ingrowth [22]. Long et al. reported the primary Tritanium cup required revision despite secondary screw fixation. They reported that supplemental screw fixation of the primary Tritanium cup may not decrease the risk of implant failure [25]. In our cases with screw fixation, there were no significant differences in the appearance of radiolucent lines in the Tritanium cup with or without screws.

This study had several limitations. First, the follow-up period was relatively short. Second, this was not a randomized clinical trial. Third, this was a retrospective study. Fourth, because there were three primary surgeons and the selection of cups, reaming method, and whether screws were used were at the surgeon’s discretion, the possibility of selection and surgeon bias has to be considered in this study. Fifth, we only used postoperative anteroposterior radiography of the pelvis to analyze the presence of radiolucent lines. Oishi et al. assessed radiolucent lines around highly porous titanium cups (Tritanium) using digital tomosynthesis at the 2-year follow-up. They reported that digital tomosynthesis detected more radiolucent lines around the Trident cups than plain radiography [26]. Analysis using a high-resolution device, such as digital tomosynthesis, may allow for more precise evaluation. Finally, the number of enrolled patients was small. Despite these limitations, a significant difference was observed in the incidence of radiolucent lines around the cups. The presence of radiolucent lines suggests the potential for future cup loosening and revision; therefore, mid- or long-term follow-up should be conducted.

## 5. Conclusions

In conclusion, radiolucent lines were significantly more frequent in the highly porous titanium cup than in the HA-coated porous titanium cup after primary THA at the 2-year follow-up. The results of this study suggest that, compared to the three-dimensional structure of porous titanium, the hydroxyapatite coating of porous titanium had a greater influence on bone ingrowth in the short term. The meaning of these findings in the long-term is unclear yet.

## Figures and Tables

**Table 1:** Patient demographic data according to highly porous titanium cup and HA-coated porous titanium cup.

	Highly Porous Titanium Cup	HA-Coated Porous Titanium Cup	*p*-Value
No. of hips	17	34	
Age (y) *	63.8 ± 15.7	67.0 ± 14.9	0.47
Sex (M/F)	3/14	5/29	1
Height (cm) *	153.1 ± 10.2	152.7 ± 8.3	0.88
Weight (kg) *	56.2 ± 11.5	54.6 ± 9.7	0.61
Body mass index (kg/m^2^) *	23.9 ± 3.8	23.5 ± 4.2	0.75
Lumbar BMD (t-score) *	–1.6 ± 1.7	–0.6 ± 1.9	0.16
Diagnosis (no. of patients)			
OA	10	28	0.08
ONFH	7	5	
ISF	0	1	

* Values are expressed as mean and standard deviation. BMD, body mineral density; OA, osteoarthritis; ONFH, osteonecrosis of the femoral head; ISF, insufficiency fracture.

**Table 2:** Preoperative and postoperative results of the modified Harris hip score (HHS).

	Highly Porous Titanium Cup	HA-Coated Porous Titanium Cup	*p*-Value
Preoperative HHS *	44.5 ± 12.5	47.2 ± 10.3	0.41
Postoperative (2 years) HHS *	89.3 ± 7.4	92.3 ± 6.6	0.14

* Values are expressed as mean and standard deviation.

**Table 3:** Number of hips with radiolucent lines by DeLee and Charnley zone at the last follow-up.

DeLee and Charnley Zones	Highly Porous Titanium Cup	HA-Coated Porous Titanium Cup	*p*-Value
One zone	5 (29%) (zone I: 3, zone III: 2)	0 (0%)	
Two zones	2 (11%) (zone I & II: 1, zone I & III: 1)	0 (0%)	
Three zones	6 (35%)	0 (0%)	
Total	13/17 (76%)	0/34 (0%)	<0.001 *

* Fisher’s exact test was performed.

## Data Availability

The datasets generated and/or analyzed in the current study are available from the corresponding author upon reasonable request.
